# Supplementary material for: Experimentally realized physical-model-based frugal wave control in metasurface-programmable complex media
Source: Nat Commun. 2024 Apr 2;15:2841. doi: 10.1038/s41467-024-46916-2 (PMC10987616; doi:10.1038/s41467-024-46916-2)
Supplement: Supplementary file 1 — Supplementary Information [file 41467_2024_46916_MOESM1_ESM.pdf]

SUPPLEMENTARY INFORMATION:

Experimentally realized physical-model-based frugal wave control in metasurface-programmable complex media

Jérôme Sol, Hugo Prod'homme, Luc Le Magoarou, and Philipp del Hougne*

Univ Rennes, INSA Rennes, CNRS, IETR-UMR 6164, F-35000 Rennes, France

* Correspondence to philipp.del-hougne@univ-rennes.fr

CONTENTS

Figure S1. Normal-incidence characterization of the programmable metasurface 2

Figure S2. Physical-model-based coherent wave control involving scattering coefficients that were not included in the calibration data 3

Figure S3. Calibration of physical model for a real-life indoor setting 4

Figure S4. Impact of inaccurate channel estimation and noise on the lower bound of a SISO wireless channel's mutual information 5

Supplementary Note 1. Paradigms for Tailoring Wave-Matter Interactions 6

Supplementary Note 2. Forward Model, Inverse Model, Inverse Design 8
A. Forward Model 8
B. Inverse Model 8
C. Inverse Design 9
D. Summary 10

Supplementary Note 3. Formulation of Physics-Based Model 11
A. Overview 11
B. Our Key Contributions Are Beyond Model Formulation 11
C. Polarizability-Based Model Reduced to Primary Entities 12
D. Example with Closed-Form Background Green's Function 14
E. Summary 15

Supplementary Note 4. Programmable Metasurface Prototype 16

References 17

Figure S1. NORMAL-INCIDENCE CHARACTERIZATION OF THE PROGRAMMABLE METASURFACE

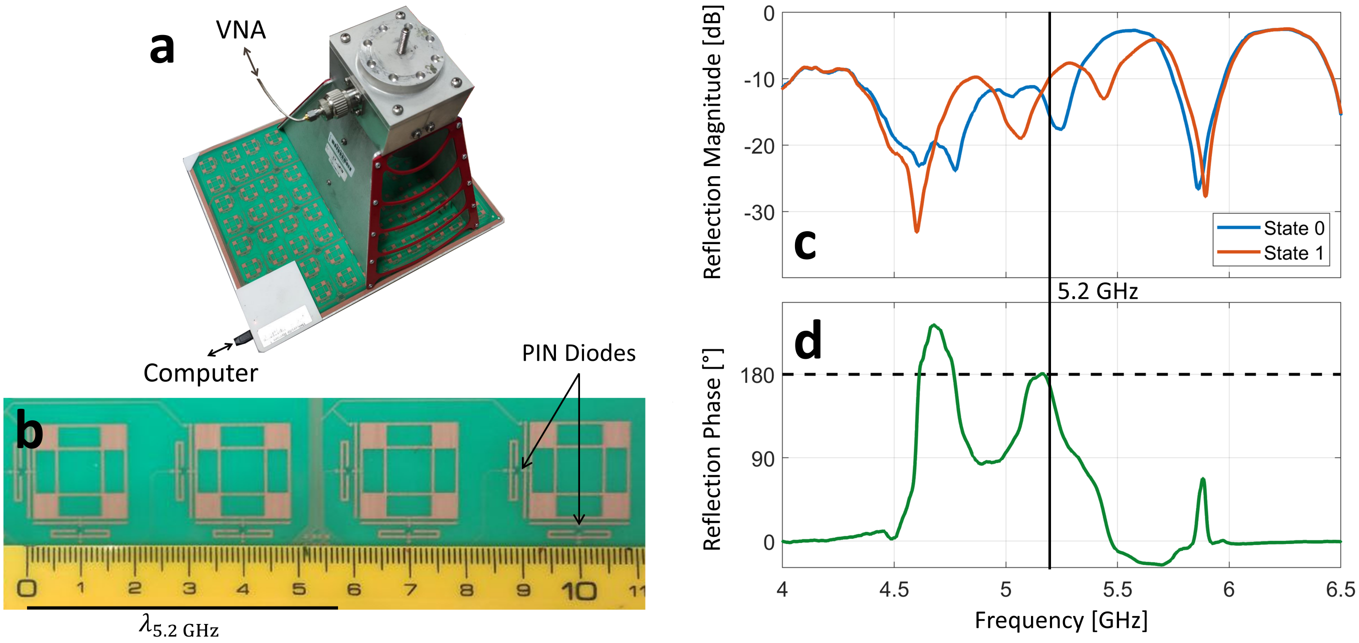

FIG. S1. **Normal-incidence characterization of the programmable metasurface. a,** Measurement setup involving a horn antenna and the programmable metasurface. **b,** Close-up view of four meta-atoms with a scale bar. Each programmable meta-atom contains two PIN diodes that enable independent control over the horizontally and vertically polarized electric field components. **c-d,** Magnitude (**c**) and phase (**d**) of the reflection coefficient measured with the setup shown in **a** when all meta-atoms are in their '0' (blue) or '1' (red) state. Close to our working frequency of 5.2 GHz, the reflection coefficients of the two possible states differ by roughly 180° in phase and have roughly the same magnitude.

Figure S2. PHYSICAL-MODEL-BASED COHERENT WAVE CONTROL INVOLVING SCATTERING COEFFICIENTS THAT WERE NOT INCLUDED IN THE CALIBRATION DATA

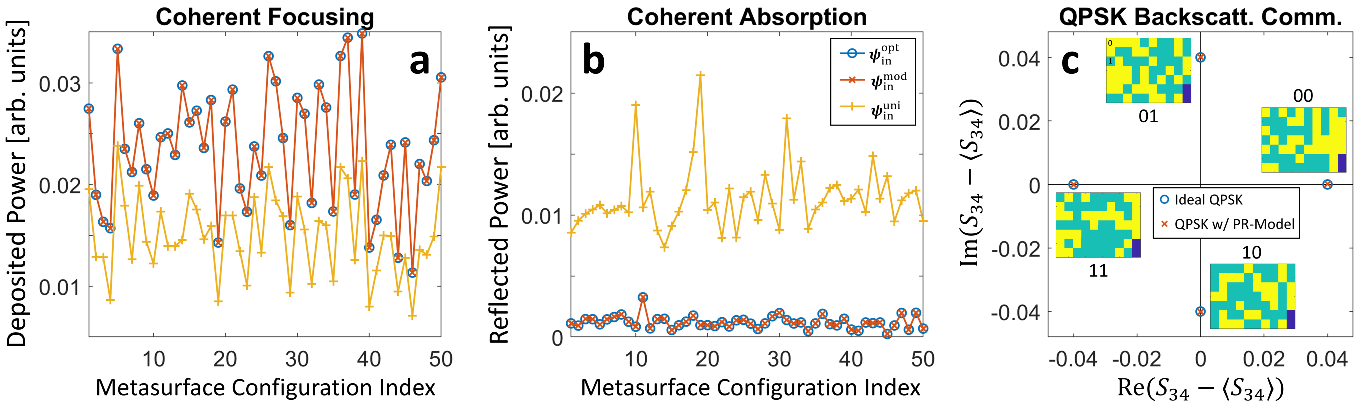

FIG. S2. **Physical-model-based coherent wave control involving scattering coefficients that were not included in the calibration data (after correction of the systematic offset).** Information about S_{33}, S_{34}, S_{43}, and S_{44} was excluded from the data used to calibrate the GFR-model used for wave control in this figure. Hence, to specifically test the wave control involving the unseen scattering coefficients, we choose to coherently focus energy on port 3 (rather than port 1 as in Fig. 3) in **a** and to base our QPSK backscatter communications on S_{34} (rather than S_{24} as in Fig. 3) in **c**. **a,** Deposited energy at port 3 upon injecting a coherent wavefront through the remaining three ports (for 50 random unseen metasurface configurations). $\psi_{\text{in}}^{\text{opt}}$ (blue) is the benchmark (and provably optimal) wavefront obtained via phase conjugation given perfect knowledge of the relevant scattering coefficients, $\psi_{\text{in}}^{\text{mod}}$ (red) is obtained with the same approach but using the GFR-Model, and $\psi_{\text{in}}^{\text{uni}}$ (yellow) is a uniform wavefront (see Methods for details). Our GFR-Model achieves on average 99.99 % of the ideal focusing efficiency with the ground-truth complex-valued transmission vector in this example, whereas an arbitrary input wavefront (e.g., a uniform one) achieves only 79.10 % of the ideal focusing. By selecting the best metasurface configuration out of the 10^5 ones used for calibration, we achieve a deposited energy of 0.0494 arb. units using our GFR-Model, compared to 0.0499 arb. units with ideal ground-truth knowledge. **b,** Reflected power upon injecting a coherent wavefront through all four ports (for 50 random unseen metasurface configurations). High absorption corresponds to low reflected power. $\psi_{\text{in}}^{\text{opt}}$ (blue) is the benchmark (and provably optimal) wavefront obtained via an eigendecomposition of $\mathbf{S}^\dagger\mathbf{S}$ assuming perfect knowledge of $\mathbf{S}$, $\psi_{\text{in}}^{\text{mod}}$ (red) is obtained with the same approach but using the GFR-Model, and $\psi_{\text{in}}^{\text{uni}}$ (yellow) is a uniform wavefront (see Methods for details). If we jointly optimize the metasurface configuration and the wavefront by selecting the most suitable metasurface configuration out of the 10^5 ones used for calibration, our GFR-Model points toward the same metasurface configuration as the ideal ground-truth knowledge, such that both achieve a minimal reflected power of -42.0 dB in this example. **c,** Identification using the GFR-Model of four metasurface configurations (shown as insets) that enable quadrature-phase-shift-keying (QPSK) backscatter communications when port 3 radiates a continuous-wave signal and port 4 detects the received phase (or vice versa) (see Methods for details).

Figure S3. CALIBRATION OF PHYSICAL MODEL FOR A REAL-LIFE INDOOR SETTING

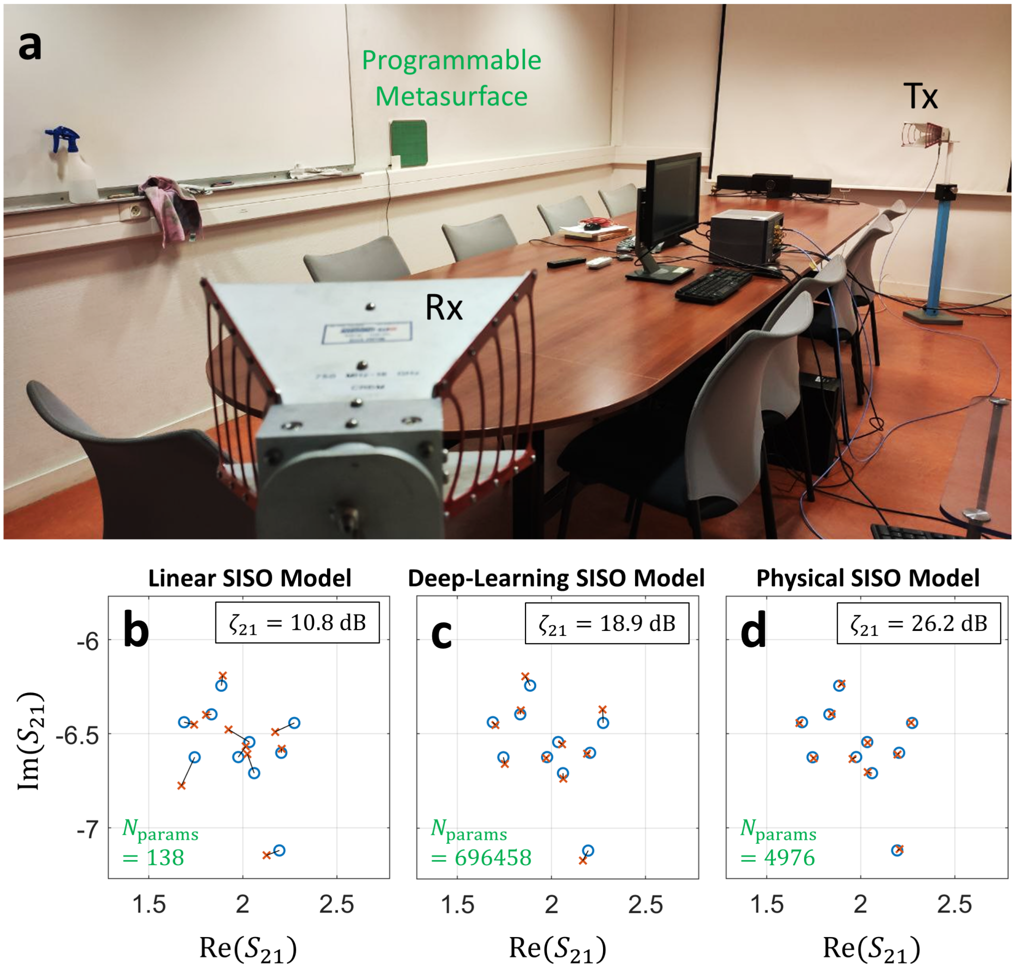

FIG. S3. **Calibration of physical model for a real-life indoor setting. a,** Photographic image of the indoor setting (a 6.57m × 3.18m × 2.49m meeting room) featuring the programmable metasurface and two horn antennas pointing toward the metasurface. **b-d,** Measured ground truth (blue circle) and model predictions (red cross) for the transmission between the two antennas (for ten random unseen metasurface configurations) for the linear model (**b**), the deep-learning model (**c**), and the physical model (**d**). The achieved accuracy ζ_{21} and number of model parameters N_{params} are indicated.

Figure S4. IMPACT OF INACCURATE CHANNEL ESTIMATION AND NOISE ON THE LOWER BOUND OF A SISO WIRELESS CHANNEL'S MUTUAL INFORMATION

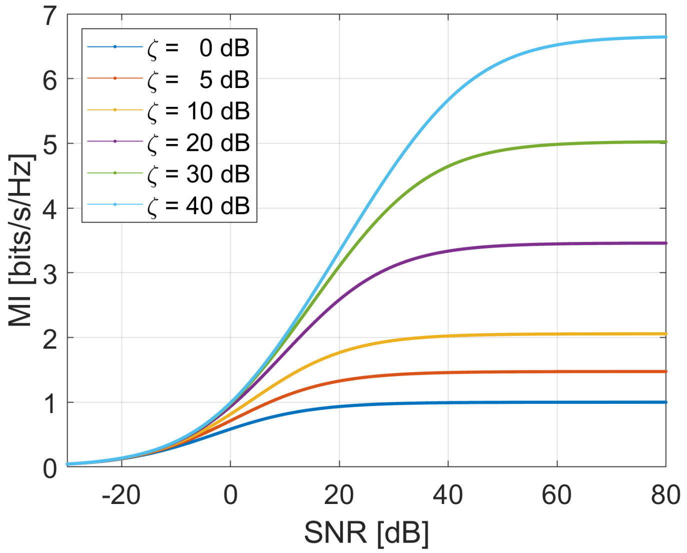

FIG. S4. **Impact of inaccurate channel estimation (quantified by ζ) and noise (quantified by SNR) on the lower bound of the mutual information (MI) of a SISO wireless channel.** In the low-SNR regime, the noise dominates and the curves for different ζ overlap. In the high-SNR regime, ζ dominates and the curves asymptotically approach limits imposed by the value of ζ. The lower bound on the MI heavily depends on ζ in the high-SNR regime. The curves are computed with $\mathrm{MI} = \log_2\left(1 + \frac{1}{\frac{1}{\mathrm{SNR}} + \frac{1}{\zeta}}\right)$ [1].

Supplementary Note 1. PARADIGMS FOR TAILORING WAVE-MATTER INTERACTIONS

We distinguish between three qualitatively different paradigms for controlling linear wave-matter interactions:

1. **Metamaterial Engineering.** The entire scattering system is conceived from scratch (within bounds imposed by physical laws and fabrication constraints) in order to achieve a desired wave-matter interaction.

2. **Wavefront Shaping.** The scattering system is static and the incident wavefront is coherently generated in order to achieve a desired wave-matter interaction.

3. **Tuning Complex Media In Situ.** The scattering system is a complex rich-scattering medium that includes tunable degrees of freedom (e.g., a programmable metasurface inside a chaotic cavity). The tunable degrees of freedom are configured in order to achieve a desired wave-matter interaction.

To formalize these paradigms and to clarify their qualitative differences, let us consider an N_{A}-port linear wave system that is described by the linear input-output relation

$$\mathbf{y} = \mathbf{S}\mathbf{x}, \tag{S1}$$

where $\mathbf{x} \in \mathbb{C}^{N_{\mathrm{A}} \times 1}$ and $\mathbf{y} \in \mathbb{C}^{N_{\mathrm{A}} \times 1}$ are the input and output wavefronts, respectively, and $\mathbf{S} \in \mathbb{C}^{N_{\mathrm{A}} \times N_{\mathrm{A}}}$ denotes the system's scattering matrix.

1. In metamaterial engineering, the entire $\mathbf{S}$ is designed (within bounds).

2. In wavefront shaping, the entire $\mathbf{x}$ is designed (within bounds).

3. In tuning a complex medium in situ, only one part of the diagonal of an interaction matrix is designed, and $\mathbf{S}$ is one block of the inverse of that interaction matrix. (See rigorous formulation in Supplementary Note 3 C.)

Of these three paradigms, the last two are confronted with unknown complex media and involve in situ control over either the impinging wavefront (in the case of wavefront shaping) or the metasurface configuration (in the case of tunable complex media). Therefore, given the unknown complex medium, two classes of wave control can be distinguished here:

1. **Closed-loop wave control.** An algorithm iteratively adjusts the controllable parameters (input wavefront or metasurface configuration) *based on feedback measured experimentally in situ after each iteration* to assess the extent to which the current wave-matter interaction resembles the targeted one, in order to then adjust the controllable parameters in the next iteration with the goal to increase the resemblance with the targeted wave-matter interaction.

2. **Open-loop wave control.** Based on a previously calibrated forward model mapping the controllable parameters (input wavefront or metasurface configuration) to the resulting wave-matter interaction, an algorithm optimizes the setting of the controllable parameters *without any additional experimental in situ measurements*. The resulting wave-matter interaction for any conceivable setting of the controllable parameters can be accurately predicted with the calibrated forward model without requiring further access to and feedback from the experiment.

To optimize the controllable parameters for a new targeted wave-matter interaction, additional measurements are hence required in closed-loop wave control whereas no additional measurements are required in open-loop wave control.

Wavefront shaping for complex media was initially proposed based on iterative in situ optimizations in Ref. [2]. These iterative in situ optimization were of closed-loop nature, i.e., any new wave control functionality would require new measurements. Once Ref. [3] reported the measurement of a complex medium's transmission matrix, open-loop wave control was enabled: all possible experiments could be simulated on a computer based on a given system's measured transmission matrix, and thus no new measurements were required for a new wave control functionality. The transmission matrix also unlocked a wealth of operator-based wave control protocols. Later, efforts to estimate the complex-valued transmission-matrix with intensity-only data (i.e., without any phase measurements) were reported [4–6].

The field of in situ tunable complex media was similarly initiated based on iterative in situ optimizations in Refs. [7, 8]. However, the field has to date not gone beyond this stage and remains (prior to our work presented in the main text) limited to closed-loop optimizations. Our results in the first half of our main text (Fig. 1 and Fig. 2) validate experimentally a physical-model-based open-loop approach. Thereby, the first half of our main text

(Fig. 1 and Fig. 2) plays a role for in situ tunable complex media that is comparable to the role of Ref. [3] in the field of wavefront shaping. Then, Fig. 3 in our main text reports the phaseless estimation of the physical-model parameters, enabling open-loop wave control in tunable complex media without ever measuring phase. The role of this achievement for in situ tunable complex media is comparable to the role of Refs. [4–6] and references therein in the field of wavefront shaping. Then, Fig. 4 in our main text reports the parameter estimation for our physics-based model without ever measuring a subset of the scattering coefficients. This achievement does not have an analogue in the field of wavefront shaping.

We recall at this stage that the physics of wavefront shaping and in situ tunable complex media is fundamentally different: in the former the complex medium is simply treated as a linear black box and one has control over the incident wavefront $\mathbf{x}$ whereas in the latter one has some control over knobs inside the black box (specifically, control over a part of the diagonal of the interaction matrix, see Supplementary Note 3 C). In particular, this means that obtaining a calibrated forward model to achieve open-loop wave control is much more challenging in the case of in situ tunable complex media than it is in the case of wavefront shaping, because it involves a non-linear mapping in the former but a linear mapping in the latter case.

The second half of our manuscript unlocks **previously unimagined** and **previously inaccessible** regimes of **frugal channel estimation and coherent wave control** in metasurface-programmable complex media. Indeed, neither the closed-loop optimizations of metasurface configurations in complex media nor open-loop neural surrogate forward models would be able to achieve coherent wave control without any phase measurement or without any measurement of a subset of the involved scattering coefficients.

Supplementary Note 2. FORWARD MODEL, INVERSE MODEL, INVERSE DESIGN

In this section, we clarify the conceptual differences between (i) a forward model, (ii) an inverse model, and (ii) inverse design. This section does not attempt to offer a thorough literature review but instead only seeks to clarify the key high-level concepts. Recent reviews [9, 10] provide in-depth discussions of these concepts within the context of metamaterial engineering (as opposed to tuning a complex medium in situ). We pay special attention to mentioning specifically works from the literature on metasurface-programmable complex media.

A. Forward Model

A forward model is a function $\mathcal{F}$ that maps the configuration of a system (in our case defined by the metasurface configuration $\mathbf{c}$) to its transfer function (in our case the scattering matrix $\mathbf{S}$ or a block thereof):

$$\mathbf{S} = \mathcal{F}(\mathbf{c}). \tag{S2}$$

To map a given metasurface configuration $\mathbf{c}$ to the corresponding scattering matrix $\mathbf{S}$, there are in principle four approaches [10]:

1. **Theoretical Explicit Mathematical Model (TEMM).** TEMM refers to an exact mathematical expression, derived ultimately from Maxwell's equations, that predicts the scattering matrix for a given configuration in a given experimental setting. Our work in the main text presents the first TEMM to describe a metasurface-programmable complex medium of *unknown* geometry and material properties.

2. **Computational Electromagnetic Simulation (CEM).** CEM refers to numerically solving Maxwell's equations for the specific system configuration of interest. In the case of metasurface-programmable complex media, CEM is typically not viable because (i) the system is many wavelengths large, (ii) the exact geometry of the system is unknown, and (iii) the exact material composition of the system is unknown.

3. **In Situ Experiment (ISE).** ISE refers to experimentally tuning the metasurface to the configuration of interest in the setting of interest, and measuring in situ the scattering matrix (for example, using a vector network analyzer in our case). In the case of metasurface-programmable complex media, ISE has to date been the only viable route, given the lack of a TEMM (prior to our present paper) and the unfeasibility of CEM.

4. **Learned Surrogate Neural Forward Model (LSNFM).** In the absence of a TEMM, and when CEM is too costly or unfeasible (as in our case), one can train a surrogate neural forward model to approximate $\mathcal{F}$ based on a large set of calibration examples $\{\mathbf{c}, \mathbf{S}(\mathbf{c})\}$ obtained via ISEs. The term "digital twin" usually refers to LSNFMs.

Remark: *The models proposed in Refs. [11–13] constitute a TEMM only in the abstract (unrealistic) case in which the scattering environment is composed of discrete dipoles of known properties surrounded by free space. This is clearly not the case in the experimental reality of metasurface-programmable complex media tackled in our work. Our complex medium is composed of continuous extended objects of unknown geometry and unknown material properties.*

Within the context of metasurface-programmable complex media, no attempts at learning surrogate neural models for a specific experimental setting were reported to date. However, Ref. [14] learned a surrogate neural forward model for the mapping from metasurface configuration to the second-order moments of the wireless channel (evaluated across random metasurface configurations) based on physics-compliant data that was numerically generated for an abstract (unrealistic) scattering environment composed of discrete dipoles of known properties surrounded by free space (based on Ref. [11]).

B. Inverse Model

An inverse model enables the backward mapping from transfer function to system configuration:

$$\mathbf{c} = \mathcal{F}^{-1}(\mathbf{S}). \tag{S3}$$

Unfortunately, inverse problems are often ill-posed. Multiple configurations might yield the same specified transfer function (for the specified frequency points and ports), or there may be no configuration at all that yields the specified transfer function. These are the "uniqueness" and "existence" concerns for inverse models [10].

As an aside, we note that sensing problems (imaging, localization, detection, recognition, etc.) require an inverse model to map observations (transfer function measurements) to the configuration that gave rise to them. In the sensing context, "configuration" refers more generally to anything determining the scattering system's structure, e.g., the position of a moving object, the posture of a human, etc. For a measured transfer function, there is no "existence" concern, and the "uniqueness" concern is usually alleviated by adding a sufficient number of frequency points or ports to make the transfer function observation unique for any given configuration.

Within the realm of metasurface-programmable chaotic cavities, various attempts at learning surrogate neural inverse models have been made in such sensing contexts, i.e., a neural network has been trained with a large amount of examples to approximate the mapping of $\mathcal{F}^{-1}$. A common approach is to use frequency diversity (i.e., measuring the transfer function over a large frequency range) to avoid the uniqueness concern and unambiguously identify the position of an object [15] or the configuration of a programmable metasurface [16]. If a programmable metasurface is present, instead of using it as the "scene" that is to be "imaged", the programmable metasurface can also serve as a mechanism that generates measurement diversity to achieve uniqueness without many frequency points or ports. For instance, the metasurface can be deployed with a fixed series of random configurations to achieve sufficiently diverse transfer function observations, and hence uniqueness for a localization problem could be guaranteed despite measuring at a single frequency and with a single port [15]. Interestingly, in both cases [15, 16], (deeply) sub-wavelength sensing without accessing any evanescent waves is achieved because the chaotic cavity acts like a generalized interferometer (see discussion in Ref. [15]).

C. Inverse Design

Inverse design is the main motivation behind efforts to obtain forward models and/or inverse models. Inverse design is concerned with identifying a configuration whose corresponding transfer function satisfies as closely as possible some objective. In our case, a cost function $\mathcal{C}(\mathbf{S}(\mathbf{c}))$ quantifies to what extent the scattering matrix corresponding to a given metasurface configuration satisfies the objective. The considered objectives in our main text are (i) maximizing the focused intensity, (ii) maximizing the absorbed energy, and (iii) maximizing the resemblance of the constellation diagram with the ideal QPSK constellation diagram. In practice, objectives are usually formulated as a minimization problem (e.g., maximizing the absorbed energy is equivalent to minimizing the outgoing energy) and, in addition, the possible configurations are constrained (in our case to be binary). The inverse design problem is hence formulated as follows:

$$\min_{\mathbf{c}} \mathcal{C}(\mathbf{S}(\mathbf{c})) \tag{S4a}$$

$$\text{s.t. } [\mathbf{c}]_i \in \{\alpha_0, \alpha_1\}. \tag{S4b}$$

In order to solve this optimization problem, a plethora of approaches has been proposed of which we mention only a few here. The interest reader is referred to Refs. [9, 10] for more background on solving inverse design problems in the context of metamaterial engineering (rather than optimizing tunable complex media).

If one aims to perform inverse design based on a forward model $\mathcal{F}$, then one can, for instance, (i) use iterative gradient descent algorithms (making use of $\mathcal{F}$ at every iteration), or (ii) use $\mathcal{F}$ to generate a very large dictionary (composed of pairs of configurations and corresponding transfer functions) that can then be searched for the one yielding the lowest cost function, or (iii) use adjoint methods that back-propagate errors and can be made compatible with binary constraints via tricks like the temperature parameter used in Ref. [17]. Note that this list is not exhaustive. In Ref. [14], a neural surrogate forward model was used in combination with a genetic algorithm to identify an optimized RIS configuration (only based on physics-compliant numerically generated data, without experiments). In our work presented in the main text, we use a physics-based model in combination with the mentioned dictionary search method. However, our contribution is not the inverse design algorithm. We merely use a simple example of an inverse design algorithm to demonstrate that our calibrated model enables highly accurate coherent wave control (even without ever measuring phase information or certain scattering coefficients).

If one aims to perform inverse design based on an inverse model $\mathcal{F}^{-1}$, one has to deal with the uniqueness and existence concerns of inverse models. Moreover, the design space may be discontinuous [10]. Many ideas for how to make use of $\mathcal{F}^{-1}$ in inverse design have been proposed in the recent literature, including tandem networks, autoencoders, generative adversarial networks, etc. [9, 10]. So far, inverse design based on an inverse model has not been reported for metasurface-programmable complex media. A neural surrogate inverse model was trained in Ref. [16] but no attempt at using it for inverse design was reported. It is questionable whether such a neural surrogate inverse model could be directly used to identify, for instance, a metasurface configuration enabling coherent perfect absorption (CPA) without having addressed the one-to-many problem, as illustrated in Box 2 in Ref. [9] and explained in Sec. 5.1 of Ref. [10].

Irrespective of the chosen inverse design approach, there is generally no guarantee that the optimized metasurface configuration is globally optimal. However, heuristic evidence (e.g., in Ref. [17]) shows that the optimization space typically contains many local optima of comparable quality, such that different runs with the same inverse design algorithm yield different optimized configurations corresponding roughly to similar cost functions. Of course, in the case of the dictionary search approach that we use in the main text, running the same algorithm twice on the same dictionary will yield exactly the same optimized configuration in both runs.

D. Summary

Within the context of metasurface-programmable complex media, our work reports the first calibration of a theoretical explicit mathematical forward model to a setting of unknown geometry and material properties, enabling open-loop wave control within the emerging paradigm of metasurface-programmable unknown complex media for the first time. Based on this forward model, we perform inverse design with a dictionary search approach in order to illustrate open-loop physical-model-based coherent wave control. Moreover, we demonstrate open-loop coherent wave control in this setting without ever measuring phase or without ever measuring some of the involved scattering coefficients, thereby unlocking previously unimagined regimes of frugal coherent wave control that are inaccessible via closed-loop wave control or using neural surrogate forward models.

Supplementary Note 3. FORMULATION OF PHYSICS-BASED MODEL

A. Overview

In this supplementary note, we detail the formulation of our physics-based model and explain in depth how it relates to the recent literature on end-to-end metasurface-parametrized channel models for rich-scattering conditions [11–13]. Although we do refine the model formulation with respect to these references in two aspects (detailed below), the key contributions of our work do *not* relate to the model formulation (see Supplementary Note 3 B below).

Prior to our work presented in the main text, it was generally believed that the overwhelming complexity of an unknown experimental chaotic cavity implies that the mapping from metasurface configuration to scattering coefficients could be captured, if at all possible, only using deep-learning approaches. Although the recent Refs. [11–13] propose models of metasurface programmable rich-scattering channels, these models consider abstract scattering environments composed of *discrete* dipoles surrounded by free space. Whether it is feasible to describe a specific unknown experimental environment (unknown geometry and unknown material properties) composed of *continuous* extended scattering objects with a tractable number of discrete dipoles as in Refs. [11–13] was unclear, as was the question of how to identify these dipoles' characteristics.

In our work, **we do not even attempt to explicitly model the scattering environment**. Instead, using the insight that the scattering environment's influence is merely to modify the coupling between the primary wireless entities (antennas and meta-atoms), we work directly with a representation of the system reduced to these primary entities. Therein, the *background* Green's functions lump together the coupling between the primary wireless entities due to proximity and reverberation – in contrast to, for example, Ref. [11] in which the *free-space* Green's function was used, i.e., assuming free space as the background. For our purpose of accurately mapping the metasurface configuration to the scattering coefficients, there is at no point a need to separate the different contributions to the background Green's functions, nor would this generally be even possible without ambiguity.

B. Our Key Contributions Are Beyond Model Formulation

Before detailing our physics-based model formulation and how we have refined previous formulations proposed in Refs. [11–13], we would like to stress in this section that the key contributions of our work presented in the main text do *not* relate to the model formulation but are as follows:

Our work introduces open-loop physical-model-based wave control in metasurface-programmable complex media. Thereby, along the way, our work not only validates the polarizability-based physics-compliant channel model experimentally for the first time, but it also provides the first methodology to perform physics-compliant end-to-end channel estimation in metasurface-parametrized environments. Most interestingly, our work discovers that physics-compliant channel estimation overcomes a plethora of issues plaguing existing channel estimation schemes. Specifically, **our work identifies and experimentally validates new techniques for frugal channel estimation**. In particular, our work discovers that by using physics-compliant channel estimation, it is possible to

1. benefit from a favorable dependence of the amount of required calibration examples on the number of wireless channels (i.e., fewer examples are needed the more channels are to be estimated). [See Fig. 2 in the main text.]
2. perform metasurface-parametrized-channel estimation without phase information, i.e., purely based on non-coherent measurements. [See Fig. 3 in the main text.]
3. perform metasurface-parametrized channel estimation without any calibration examples for some of the channels of interest. [See Fig. 4 in the main text.]

These frugal channel estimation methods were **previously unimagined and previously inaccessible**, and they are of **high technological relevance for the smart radio environments envisioned as pivotal ingredient of next-generation wireless networks**. These surprising (i.e, unexpected) discoveries enable us to report

1. an orders of magnitude more accurate and orders of magnitude more compact forward model calibrated with orders of magnitude fewer calibration examples (in one case only ≈ 400 out of the $2^{68} \approx 3 \times 10^{20}$ possible metasurface configurations), compared to the benchmarks (linear and deep learning approaches).
2. the first experimental demonstration of coherent wave control in a metasurface-parametrized rich-scattering system without ever measuring phase, especially for the very timely coherent perfect absorption (CPA) example. CPA is inherently a coherent phenomenon, and we can tune a system to CPA and observe CPA without ever measuring phase.

3. the first experimental demonstration of coherent wave control in a metasurface-parametrized rich-scattering system based on channels that were never measured, especially for the QPSK backscatter communications example.

Remark: *We perform* ***open-loop*** *wave control combining (i) wavefront shaping (i.e., coherent control of the input wavefronts) and (ii) structural control (i.e., choosing the metasurface configuration)* ***based on a (physical) forward model****. Combined wavefront shaping and structural control has previously been reported, for instance, in Refs. [18–21], but without any understanding of how the scattering matrix depends on the metasurface configuration such that very long* ***closed-loop*** *iterative in situ optimizations were necessary to identify optimized metasurface configurations. Specifically, at every iteration, a new in situ experimental measurement was necessary. Most importantly, performing this wave control without ever measuring phase (as we do in Fig. 3 in the main text) or without ever measuring some of the utilized scattering coefficients (as we do in Fig. 4 in the main text) was certainly out of reach and had not been imagined to be possible.*

Our above-listed discoveries are of substantial technological significance since they alleviate the burden of channel estimation in ways that have not been imagined possible prior to our work:

1. massive reduction (instead of increase) of the number of required calibration examples as more channels are of interest, as is the case for massive MIMO, for instance.

2. total removal of the requirement for coherent detection, i.e., phase measurements. Non-coherent measurements do not require synchronization and are significantly less costly in terms of hardware.

3. removal of the requirement to have calibration examples for all channels of interest, again reducing the measurement burden for channel estimation. This is particularly relevant, for example, to settings involving antennas that can only operate in receiving mode.

Besides these key contributions of our work, we have also improved the formulation of the polarizability-based model proposed in Refs. [11, 12] by extending it from scattering environments composed of discrete dipoles to scattering environments composed of continuous extended scattering objects, as well as by extending it from 2D to 3D. An equivalent impedance-based model in Ref. [13] was limited to discrete dipoles similarly to Refs. [11, 12]. However, these refinements of the model formulation are not the key contributions of our work presented in the main text. Our key contributions were summarized in the previous paragraphs in this Supplementary Note 3 B.

C. Polarizability-Based Model Reduced to Primary Entities

In this section, we provide a more detailed derivation of the utilized physical model that is briefly summarized in the section titled "Physical Model" in the main text (see also Sec. 2.1.1 in Ref. [22]).

Our primary wireless entities of interest are N_A antennas (of which N_T are transmitting antennas and the remaining $N_\mathrm{R} = N - N_\mathrm{T}$ are receiving antennas) as well as the N_S programmable meta-atoms. All primary entities are hence naturally discrete. We model each of these N entities as a dipole. The ith dipole is characterized by its polarizability (α_i) that relates its induced dipole moment (p_i) to the total incident field on the dipole along the dipole's orientation (E_i) :

$$p_i = \alpha_i E_i. \tag{S5}$$

Polarizability is a local concept, i.e., the polarizability of the ith dipole depends in no way on what happens anywhere in the scattering system except for the location of the ith dipole. For instance, if the scattering system is perturbed somewhere away from the ith dipole, the value of α_i does not change. All dipoles representing antennas are nominally identical and static, such that we assume the same polarizability α_A for all of them. All dipoles representing meta-atoms are nominally identical, and each meta-atom can be individually configured to be in one of two possible states, such that we assume that the polarizability of the meta-atoms is either α_0 or α_1, depending on the metasurface configuration. The latter is summarized by the vector $\mathbf{c} \in \mathbb{C}^{N_\mathrm{S}\times 1}$ which contains the N_S polarizability values of the meta-atoms. Because the configuration of a meta-atom determines a local property of the scattering system, this polarizability formulation appears to be the most compact/transparent approach. However, equivalent formulation in terms of impedance are possible [13].

The field incident on the ith dipole is the superposition of the incoming wavefront (which is non-zero only for the N_A dipoles representing antennas) and the fields re-radiated by the dipoles:

$$E_i = E_i^{\mathrm{ext}} + \sum_{j=1}^{N} G_{ij} p_j. \tag{S6}$$

G_{ij} is the *background* Green's function between the positions of the ith and jth dipole. It is reciprocal, i.e., $G_{ij} = G_{ji}$, and non-local, meaning that it depends on the entire background scattering system and any perturbation therefore, no matter where in space, will impact the value of G_{ij}. Importantly, we thus do *not* work with the free-space Green's function, as in Refs. [11, 13] where the scattering environment was assumed to be composed of discrete dipoles surrounded by free space (see Supplementary Note 3 D for details on this special case). In fact, we do not make any assumption about the nature of the background scattering system, e.g., as to whether it is composed of discrete or continuous scattering objects. In free space, $G_{ii} = 0 \; \forall \; i$; by contrast, in a non-trivial scattering medium, there are in general paths from the ith dipole back to the ith dipole that bounce around the environment without encountering any of the other dipoles. Such self-interactions are non-local whereas polarizability is a local concept, hence these self-interactions cannot be absorbed into the polarizability definition and instead result in a non-zero G_{ii}. In the case of a scattering environment composed of dipoles surrounded by free space, a closed-form expression for the background Green's functions' dependence on the properties of the environmental dipoles can be derived, as shown in Supplementary Note 3 D below.

To self-consistently determine the values of the dipole moments for a given incoming wavefront, we insert Eq. (S5) into Eq. (S6) and summarize the set of N equations in matrix form:

$$\mathbf{W}\mathbf{p} = \mathbf{E}^{\text{ext}}, \tag{S7}$$

where $\mathbf{W} \in \mathbb{C}^{N \times N}$ with

$$W_{ii} = \alpha_i^{-1} - G_{ii}, \tag{S8a}$$

$$W_{ij} = W_{ji} = -G_{ij} = -G_{ji}, \tag{S8b}$$

$\mathbf{p} = [p_1, p_2, \ldots, p_N]$ and $\mathbf{E}^{\text{ext}} = [E_1^{\text{ext}}, E_2^{\text{ext}}, \ldots, E_N^{\text{ext}}]$. Let us denote by $\mathcal{A}$ the indices of dipoles representing antennas, and by $\mathcal{S}$ the remaining indices of dipoles representing meta-atoms. Then, we can summarize the fact that external fields are only incident on dipoles representing antennas as $E_i^{\text{ext}} = 0 \; \forall \; i \in \mathcal{S}$.

Solving Eq. (S7) for $\mathbf{p}$, we obtain

$$\mathbf{p} = \mathbf{W}^{-1}\mathbf{E}^{\text{ext}}. \tag{S9}$$

Here, the matrix inversion compactly and self-consistently captures the infinite number of paths involving increasingly many bounces, as can be seen by expressing the matrix inverse as an infinite sum of matrix powers [23].

How does the interaction matrix $\mathbf{W}$ depend on the metasurface configuration $\mathbf{c}$? As stated previously, the metasurface configuration contains the inverse polarizability values of the meta-atoms. We can hence write

$$\mathbf{W} = \mathbf{A} + \mathbf{G}, \tag{S10}$$

where

$$\mathbf{A} = \begin{bmatrix} \alpha_{\text{A}}^{-1}\mathbf{I}_{\mathcal{A}\mathcal{A}} & \mathbf{0}_{\mathcal{A}\mathcal{S}} \\ \mathbf{0}_{\mathcal{S}\mathcal{A}} & \text{diag}(\mathbf{c}) \end{bmatrix} \tag{S11}$$

and $\mathbf{I}_{\mathcal{A}\mathcal{A}}$ denotes the $N_{\text{A}} \times N_{\text{A}}$ identity matrix, and $\mathbf{0}_{\mathcal{A}\mathcal{S}} = \mathbf{0}_{\mathcal{S}\mathcal{A}}^T$ denotes an $N_{\text{A}} \times N_{\text{S}}$ matrix whose entries are all zero. Meanwhile, the (i, j)th entry of $\mathbf{G}$ is $-G_{ij}$.

In Fig. 1 of our main text, we have referred to $\mathbf{A}$ as containing the "local scattering properties" of the primary entities (antennas and meta-atoms), and to $\mathbf{G}$ as containing the corresponding "non-local scattering properties" that define the coupling between the primary entities. Recall that we explained earlier in this Supplementary Note 3 C why polarizability is a local concept whereas the Green's function is a non-local concept. Importantly, the metasurface configuration only impacts the lower part of the diagonal of $\mathbf{A}$. Note also that $\mathbf{G}$ is symmetric for a reciprocal system, and that its diagonal entries are in general not zero (in the special case of the scattering environment being free space, however, $\mathbf{G}$ is hollow).

The essential physics of how waves scatter in a metasurface-programmable complex medium is captured by the inverse interaction matrix, and the dependence of this wave scattering on the metasurface configuration is clarified by the above Eq. (S10) and Eq. (S11) as well as the corresponding illustration in Fig. 1 of our main text. For instance, as detailed in Ref. [23], one can work out the infinite family of increasingly long multi-bounce paths that encounter the RIS by expressing the inverse as an infinite sum of matrix powers.

The incoming wavefronts are proportional to $[\mathbf{E}^{\text{ext}}]_{\mathcal{A}}$ and the outgoing wavefronts are proportional to $[\mathbf{p}]_{\mathcal{A}}$. Therefore, the scattering matrix $\mathbf{S} \in \mathbb{C}^{N_{\text{A}} \times N_{\text{A}}}$ is equivalent to $\left[\mathbf{W}^{-1}\right]_{\mathcal{A}\mathcal{A}}$ up to some multiplicative factors and additive

terms that do not depend on the metasurface configuration. At this point, it is important to recall our goal in order to understand that we do not need to work out the multiplicative and additive terms. (In fact, even if we wanted to, it would not be possible to unambigously determine these terms.) Our goal is to predict the scattering coefficients corresponding to any given metasurface configuration. Therefore, since the multiplicative and additive terms do not depend on the metasurface configuration, they do not impact the functional dependence of the forward model on the metasurface configuration and can hence be absorbed into the parameters that must be estimated anyway for $\mathbf{W}$. Therefore, we work in our main text with

$$\mathbf{S} = \left[\hat{\mathbf{W}}^{-1}\right]_{\mathcal{A}\mathcal{A}} = \left[\left(\begin{bmatrix} \hat{\alpha}_{\mathrm{A}}^{-1}\mathbf{I}_{\mathcal{A}\mathcal{A}} & \mathbf{0}_{\mathcal{A}\mathcal{S}} \\ \mathbf{0}_{\mathcal{S}\mathcal{A}} & \mathrm{diag}(\hat{\mathbf{c}}) \end{bmatrix} + \begin{bmatrix} \hat{\mathbf{G}}_{\mathcal{A}\mathcal{A}} & \hat{\mathbf{G}}_{\mathcal{A}\mathcal{S}} \\ \hat{\mathbf{G}}_{\mathcal{S}\mathcal{A}} & \hat{\mathbf{G}}_{\mathcal{S}\mathcal{S}} \end{bmatrix}\right)^{-1}\right]_{\mathcal{A}\mathcal{A}}, \tag{S12}$$

where we use the notation $\hat{\mathbf{W}}$, $\hat{\mathbf{G}}$, $\hat{\mathbf{c}}$ and $\hat{\alpha}_{\mathrm{A}}$ instead of $\mathbf{W}$, $\mathbf{G}$, $\mathbf{c}$ and α_{A}, respectively, to denote that we have absorbed other multiplicative and additive terms into these variables. An illustration of Eq. (S12) is provided in Fig. 1 in our main text. If the antennas are split into a group of transmitting antennas (whose set of dipole indices is denoted by $\mathcal{T}$) and a group of receiving antennas (whose set of dipole indices is denoted by $\mathcal{R}$), then we can define the end-to-end wireless channel matrix $\mathbf{H} \in \mathbb{C}^{N_{\mathrm{R}} \times N_{\mathrm{T}}}$ as:

$$\mathbf{H} = [\mathbf{S}]_{\mathcal{R}\mathcal{T}} = \left[\left(\begin{bmatrix} \hat{\alpha}_{\mathrm{A}}^{-1}\mathbf{I}_{\mathcal{A}\mathcal{A}} & \mathbf{0}_{\mathcal{A}\mathcal{S}} \\ \mathbf{0}_{\mathcal{S}\mathcal{A}} & \mathrm{diag}(\hat{\mathbf{c}}) \end{bmatrix} + \begin{bmatrix} \hat{\mathbf{G}}_{\mathcal{A}\mathcal{A}} & \hat{\mathbf{G}}_{\mathcal{A}\mathcal{S}} \\ \hat{\mathbf{G}}_{\mathcal{S}\mathcal{A}} & \hat{\mathbf{G}}_{\mathcal{S}\mathcal{S}} \end{bmatrix}\right)^{-1}\right]_{\mathcal{R}\mathcal{T}}. \tag{S13}$$

The calibration of this model then simply requires the estimation of the three complex-valued local scattering parameters (i.e., α_{A}, α_0 and α_1) as well as the $(N+1)N/2$ complex-valued non-local parameters (i.e., the entries of $\mathbf{G}$ which is a symmetric matrix). This estimation is accomplished via gradient descent as detailed in the Methods section of our main text.

D. Example with Closed-Form Background Green's Function

For a generic complex medium such as the chaotic cavity from our experiments in the main text, no closed-form expression for the background Green's function exists. However, in the special case in which the scattering environment is composed of dipoles of known properties surrounded by free space, a closed-form expression for $\mathbf{G}$ can be derived. This is the case considered in Refs. [11–13] (see also Sec. 2.1.2 in Ref. [22]). Although it is an abstract (unrealistic) case, it provides valuable insights.

In this specific example, we hence consider a scenario involving N_{A} antenna dipoles, N_{S} meta-atom dipoles, and N_{E} environmental dipoles. There are two equivalent ways of looking at such a system: (i) we have $N = N_{\mathrm{A}} + N_{\mathrm{S}}$ dipoles coupled via background Green's functions that are determined by the N_{E} environmental dipoles' properties; (ii) we have $\tilde{N} = N + N_{\mathrm{E}}$ dipoles surrounded by free space that are coupled by free-space Green's functions. For free-space Green's functions, closed-form expressions exist. Because both descriptions are equivalent, the interaction matrix $\mathbf{W} \in \mathbb{C}^{N \times N}$ from (i) and the augmented interaction matrix $\tilde{\mathbf{W}} \in \mathbb{C}^{\tilde{N} \times \tilde{N}}$ from (ii) must be related as follows:

$$\mathbf{W}^{-1} = \left[\tilde{\mathbf{W}}^{-1}\right]_{\mathcal{P}\mathcal{P}}, \tag{S14}$$

where $\mathcal{P} = \mathcal{A} \cup \mathcal{S}$.

To start, we partition the augmented interaction matrix $\tilde{\mathbf{W}}$ into a 2×2 block matrix:

$$\tilde{\mathbf{W}} = \begin{bmatrix} \tilde{\mathbf{W}}_{\mathcal{P}\mathcal{P}} & \tilde{\mathbf{W}}_{\mathcal{P}\mathcal{E}} \\ \tilde{\mathbf{W}}_{\mathcal{E}\mathcal{P}} & \tilde{\mathbf{W}}_{\mathcal{E}\mathcal{E}} \end{bmatrix} = \begin{bmatrix} \mathbf{A} & \mathbf{0}_{\mathcal{P}\mathcal{E}} \\ \mathbf{0}_{\mathcal{E}\mathcal{P}} & \mathbf{A}_{\mathcal{E}\mathcal{E}} \end{bmatrix} + \begin{bmatrix} \tilde{\mathbf{G}}_{\mathcal{P}\mathcal{P}} & \tilde{\mathbf{G}}_{\mathcal{P}\mathcal{E}} \\ \tilde{\mathbf{G}}_{\mathcal{E}\mathcal{P}} & \tilde{\mathbf{G}}_{\mathcal{E}\mathcal{E}} \end{bmatrix} = \tilde{\mathbf{A}} + \tilde{\mathbf{G}}, \tag{S15}$$

where $\mathcal{E}$ denotes the set of dipole indices corresponding to the environmental dipoles. Note that $\tilde{\mathbf{G}}$ contains free-space Green's functions which are known in closed form, and that $\tilde{\mathbf{G}}$ is a hollow matrix, i.e., its diagonal entries are zero.

Applying the block matrix inversion lemma, we can now work out an expression for the background Green's function matrix $\mathbf{G}$ in terms of $\mathbf{A}_{\mathcal{E}\mathcal{E}}$ and $\tilde{\mathbf{G}}$:

$$\left[\tilde{\mathbf{W}}^{-1}\right]_{\mathcal{P}\mathcal{P}} = \left(\mathbf{A} + \tilde{\mathbf{G}}_{\mathcal{P}\mathcal{P}} - \tilde{\mathbf{G}}_{\mathcal{P}\mathcal{E}}\left(\mathbf{A}_{\mathcal{E}\mathcal{E}} + \tilde{\mathbf{G}}_{\mathcal{E}\mathcal{E}}\right)^{-1}\tilde{\mathbf{G}}_{\mathcal{E}\mathcal{P}}\right)^{-1} = \left(\mathbf{A} + \mathbf{G}\right)^{-1} = \mathbf{W}^{-1}, \tag{S16}$$

where we identify

$$\mathbf{G} = \tilde{\mathbf{G}}_{\mathcal{P}\mathcal{P}} - \tilde{\mathbf{G}}_{\mathcal{P}\mathcal{E}}\left(\mathbf{A}_{\mathcal{E}\mathcal{E}} + \tilde{\mathbf{G}}_{\mathcal{E}\mathcal{E}}\right)^{-1}\tilde{\mathbf{G}}_{\mathcal{E}\mathcal{P}}. \tag{S17}$$

While the free-space Green's function matrix $\tilde{\mathbf{G}}$ is hollow, the background Green's function matrix $\mathbf{G}$ is generally not hollow. Both $\tilde{\mathbf{G}}$ and $\mathbf{G}$ are symmetric for reciprocal systems such as our experiments in the main text. Note that $\mathbf{A}_{\mathcal{E}\mathcal{E}}$ is determined given the polarizabilities of the environmental dipoles, and $\tilde{\mathbf{G}}$ can be determined in closed form given the locations of all dipoles.

For the case of the complex medium being composed of dipoles surrounded by free space, we have hence worked out a closed-form expression for the background Green's function matrix $\mathbf{G}$. In particular, we see that its diagonal entries are generally not zero, meaning that there are self-interactions for the primary dipoles that arise due to paths that go from a given primary dipole to that same primary dipole, only bouncing of environmental dipoles along their trajectory. Such paths are non-local (they depend on the entire scattering system) and can therefore not be absorbed into the polarizability definition.

This Supplementary Note 3 D is based on Sec. III in Ref. [12]. For the sake of further contextualization, we note that similar applications of the matrix inversion lemma to obtain reduced-basis system representations were recently used to explain how hidden symmetries in non-local metamaterials enable covert scattering control [24], and similar calculations of effective wave operators were also presented within the more limited scopes of tight-binding network engineering [25] and isospectral graph reduction [26], however, without the complete scattering calculation that is shown in Ref. [24].

E. Summary

To summarize, our physics-based model used in the main text goes beyond Refs. [11, 12] that already formulated polarizability-based physics-compliant models in that the present physics-based model is generalized (and experimentally validated)

1. for continuous extended (as opposed to discrete point-like) scattering objects.

2. for 3D settings (as opposed to 2D settings).

Note that, similarly to Refs. [11, 12], the scope of Ref. [13] based on a formulation in terms of impedances rather than polarizabilities is limited to discrete point-like scattering objects.

However, albeit important, our generalization of previously formulated physics-compliant models is *not* the main contribution of the work presented in the main text. Our key contributions are explicitly listed and explained in Supplementary Note 3 B.

Supplementary Note 4. PROGRAMMABLE METASURFACE PROTOTYPE

First and foremost, we would like to stress in this Supplementary Note 4 that **the methods for estimating the parameters of a physics-based end-to-end model for metasurface-parametrized channels reported in our main text apply very generally to any type of linear programmable metasurface**, irrespective of the number of meta-atoms, the design of the meta-atoms (including their degree of programmability), and the spacing of the meta-atoms. The only restriction that we would like to point out is that the metasurface must not change the fact that the overall wave system is linear, otherwise the underlying physics fundamentally changes and a fundamentally different physics-based model would be needed.

The concept of programmable metasurfaces has been experimentally prototyped and presented under various terminologies (including tunable impedance surface, reconfigurable reflectarray, spatial microwave modulator, reconfigurable intelligent surface) since the early 2000s [27–30]. There is currently a certain mismatch between existing programmable metasurface prototypes in experimental papers and assumptions about them in many purely theoretical papers. Theoretical papers often assume that

1. there is zero coupling between the meta-atoms.
2. the meta-atoms can reflect waves with any desired reflection coefficient (for instance, unity amplitude and arbitrary phase).

In contrast, experimental papers (like our work) are usually based on programmable metasurface prototypes where

1. there is significant non-negligible coupling between the meta-atoms, even in free space.
2. the meta-atoms' programmability is restricted to 1-bit (sometimes 2-bit) to limit the control circuitry's complexity. Each meta-atom requires an individual electronic DC bias signal, and binary signals are easier to generate than continuous signals.
3. the phase and amplitude characteristics of the meta-atoms' reflection properties are moreover typically intertwined. Typical meta-atom designs are based on a Lorentzian resonator such that, for example, binary reflection coefficients of ± 1 are not realizable. As the phase changes, so does the amplitude. While it is possible to conceive meta-atoms capable of implementing arbitrary phase and amplitude control, this requires elaborate multi-resonance meta-atom designs that are not widely envisioned for RIS so far. A recent experimental demonstration of such a design can be found in Ref. [31].

There are efforts in the recent theoretical literature to make more realistic assumptions, e.g., by accounting for the intertwinement between phase and amplitude response [32, 33], or by accounting for the coupling between meta-atoms due to proximity (and sometimes also reverberation) [11–13, 34–37].

Our methods presented in the main text do not make any assumptions about the nature of the possible polarizability values. Instead, these are parameters that are estimated based on the calibration data. Because our prototype's meta-atoms are one-bit programmable, we had only two meta-atom polarizability values to estimate in the main text, but our methods would apply equally to 2-bit or multi-bit or continuously programmable meta-atoms. Moreover, our methods apply irrespective of the exact meta-atom design and spacing, since these details only impact parameters (polarizabilities and Green's functions) that we estimate anyway based on the calibration data. Our methods are furthermore in no way specific to the number of meta-atoms used in our proof-of-principle experiment which was simply determined by our available programmable metasurface prototype.

[1] M. Medard, The effect upon channel capacity in wireless communications of perfect and imperfect knowledge of the channel, IEEE Trans. Inform. Theory **46**, 933 (2000).
[2] I. M. Vellekoop and A. Mosk, Focusing coherent light through opaque strongly scattering media, Opt. Lett. **32**, 2309 (2007).
[3] S. M. Popoff, G. Lerosey, R. Carminati, M. Fink, A. C. Boccara, and S. Gigan, Measuring the transmission matrix in optics: an approach to the study and control of light propagation in disordered media, Phys. Rev. Lett. **104**, 100601 (2010).
[4] A. Drémeau, A. Liutkus, D. Martina, O. Katz, C. Schülke, F. Krzakala, S. Gigan, and L. Daudet, Reference-less measurement of the transmission matrix of a highly scattering material using a dmd and phase retrieval techniques, Opt. Express **23**, 11898 (2015).
[5] P. Caramazza, O. Moran, R. Murray-Smith, and D. Faccio, Transmission of natural scene images through a multimode fibre, Nat. Commun. **10**, 2029 (2019).
[6] J. Dong, L. Valzania, A. Maillard, T.-a. Pham, S. Gigan, and M. Unser, Phase retrieval: From computational imaging to machine learning: A tutorial, IEEE Signal Process. Mag. **40**, 45 (2023).
[7] N. Kaina, M. Dupré, G. Lerosey, and M. Fink, Shaping complex microwave fields in reverberating media with binary tunable metasurfaces, Sci. Rep. **4**, 6693 (2014).
[8] M. Dupré, P. del Hougne, M. Fink, F. Lemoult, and G. Lerosey, Wave-field shaping in cavities: Waves trapped in a box with controllable boundaries, Phys. Rev. Lett. **115**, 017701 (2015).
[9] P. R. Wiecha, A. Arbouet, C. Girard, and O. L. Muskens, Deep learning in nano-photonics: inverse design and beyond, Photonics Research **9**, B182 (2021).
[10] O. Khatib, S. Ren, J. Malof, and W. J. Padilla, Deep learning the electromagnetic properties of metamaterials—a comprehensive review, Adv. Funct. Mater. **31**, 2101748 (2021).
[11] R. Faqiri, C. Saigre-Tardif, G. C. Alexandropoulos, N. Shlezinger, M. F. Imani, and P. del Hougne, PhysFad: Physics-based end-to-end channel modeling of RIS-parametrized environments with adjustable fading, IEEE Trans. Wirel. Commun. **22**, 580 (2023).
[12] H. Prod'homme and P. del Hougne, Efficient computation of physics-compliant channel realizations for (rich-scattering) RIS-parametrized radio environments, IEEE Commun. Lett. **27**, 3375 (2023).
[13] P. Mursia, S. Phang, V. Sciancalepore, G. Gradoni, and M. Di Renzo, SARIS: Scattering aware reconfigurable intelligent surface model and optimization for complex propagation channels, IEEE Wirel. Commun. Lett. **12**, 1921 (2023).
[14] K. Stylianopoulos, N. Shlezinger, P. del Hougne, and G. C. Alexandropoulos, Deep-learning-assisted configuration of reconfigurable intelligent surfaces in dynamic rich-scattering environments, Proc. ICASSP, 8822 (2022).
[15] M. del Hougne, S. Gigan, and P. del Hougne, Deeply subwavelength localization with reverberation-coded aperture, Phys. Rev. Lett. **127**, 043903 (2021).
[16] B. W. Frazier, T. M. Antonsen Jr, S. M. Anlage, and E. Ott, Deep-learning estimation of complex reverberant wave fields with a programmable metasurface, Phys. Rev. Applied **17**, 024027 (2022).
[17] P. del Hougne, M. F. Imani, A. V. Diebold, R. Horstmeyer, and D. R. Smith, Learned Integrated Sensing Pipeline: Reconfigurable Metasurface Transceivers as Trainable Physical Layer in an Artificial Neural Network, Adv. Sci. **7**, 1901913 (2020).
[18] B. W. Frazier, T. M. Antonsen Jr, S. M. Anlage, and E. Ott, Wavefront shaping with a tunable metasurface: Creating cold spots and coherent perfect absorption at arbitrary frequencies, Phys. Rev. Research **2**, 043422 (2020).
[19] P. del Hougne, K. B. Yeo, P. Besnier, and M. Davy, Coherent wave control in complex media with arbitrary wavefronts, Phys. Rev. Lett. **126**, 193903 (2021).
[20] P. del Hougne, K. B. Yeo, P. Besnier, and M. Davy, On-demand coherent perfect absorption in complex scattering systems: time delay divergence and enhanced sensitivity to perturbations, Laser Photonics Rev. **15**, 2000471 (2021).
[21] J. Sol, A. Alhulaymi, A. D. Stone, and P. del Hougne, Reflectionless programmable signal routers, Sci. Adv. **9**, eadf0323 (2023).
[22] P. del Hougne, RIS-parametrized rich-scattering environments: Physics-compliant models, channel estimation, and optimization, arXiv:2311.11651 (2023).
[23] A. Rabault, L. Le Magoarou, J. Sol, G. C. Alexandropoulos, N. Shlezinger, H. V. Poor, and P. del Hougne, On the tacit linearity assumption in common cascaded models of RIS-parametrized wireless channels, IEEE Trans. Wirel. Commun., *in press*, arXiv:2302.04993 (2024).
[24] J. Sol, M. Röntgen, and P. del Hougne, Covert scattering control in metamaterials with non-locally encoded hidden symmetry, Adv. Mater. **TBD**, 2303891 (2023).
[25] S. Longhi, Non-Hermitian tight-binding network engineering, Phys. Rev. A **93**, 022102 (2016).
[26] L. Bunimovich and B. Webb, Isospectral transformations, Springer Monogr. Math. (2014).
[27] D. F. Sievenpiper, J. H. Schaffner, H. J. Song, R. Y. Loo, and G. Tangonan, Two-dimensional beam steering using an electrically tunable impedance surface, IEEE Trans. Antennas Propag. **51**, 2713 (2003).
[28] T. J. Cui, M. Q. Qi, X. Wan, J. Zhao, and Q. Cheng, Coding metamaterials, digital metamaterials and programmable metamaterials, Light Sci. Appl. **3**, e218 (2014).
[29] N. Kaina, M. Dupré, M. Fink, and G. Lerosey, Hybridized resonances to design tunable binary phase metasurface unit cells, Opt. Express **22**, 18881 (2014).
[30] H. Kamoda, T. Iwasaki, J. Tsumochi, T. Kuki, and O. Hashimoto, 60-GHz electronically reconfigurable large reflectarray

using single-bit phase shifters, IEEE Trans. Antennas Propag. **59**, 2524 (2011).

[31] T. Sleasman, R. Duggan, S. A. Ra'id, and D. Shrekenhamer, Dual-resonance dynamic metasurface for independent magnitude and phase modulation, Phys. Rev. Applied **20**, 014004 (2023).

[32] S. Abeywickrama, R. Zhang, Q. Wu, and C. Yuen, Intelligent reflecting surface: Practical phase shift model and beamforming optimization, IEEE Trans. Commun. **68**, 5849 (2020).

[33] H. Li, W. Cai, Y. Liu, M. Li, Q. Liu, and Q. Wu, Intelligent reflecting surface enhanced wideband MIMO-OFDM communications: From practical model to reflection optimization, IEEE Trans. Commun. **69**, 4807 (2021).

[34] G. Gradoni and M. Di Renzo, End-to-end mutual coupling aware communication model for reconfigurable intelligent surfaces: An electromagnetic-compliant approach based on mutual impedances, IEEE Wirel. Commun. Lett. **10**, 938 (2021).

[35] S. Shen, B. Clerckx, and R. Murch, Modeling and architecture design of reconfigurable intelligent surfaces using scattering parameter network analysis, IEEE Trans. Wirel. Commun. **21**, 1229 (2021).

[36] D. Badheka, J. Sapis, S. R. Khosravirad, and H. Viswanathan, Accurate modeling of intelligent reflecting surface for communication systems, IEEE Trans. Wirel. Commun. **22**, 5871 (2023).

[37] M. Akrout, F. Bellili, A. Mezghani, and J. A. Nossek, Physically consistent models for intelligent reflective surface-assisted communications under mutual coupling and element size constraint, arXiv:2302.11130 (2023).
